# Evaluating the success of community engagement in the Implementation Science Center for Cancer Control Equity

**DOI:** 10.1017/cts.2025.86

**Published:** 2025-05-16

**Authors:** Lydia E. Pace, Madeline Davies, Stephanie Martinez, Leslie Salas Karnes, Leslie Pelton-Cairns, Susan Dargon-Hart, Melissa Holguin, James G. Daly, Rebekka M. Lee, Gina R. Kruse, Karen M. Emmons

**Affiliations:** 1 Brigham and Women’s Hospital, Boston, MA, USA; 2 Massachusetts General Hospital, Boston, MA, USA; 3 Massachusetts League of Community Health Centers, Boston, MA, USA; 4 Harvard T.H. Chan School of Public Health, Boston, MA, USA; 5 University of Colorado School of Medicine, Aurora, CO, USA

**Keywords:** Community engagement, implementation science, community health centers, Clinical and Translational Science Awards, Massachusetts

## Abstract

Community-engaged research is essential to advance the implementation of evidence-based practices, but engagement quality is rarely assessed. We evaluated community health centers’ (CHCs) experiences partnering with the Implementation Science Center for Cancer Control Equity (ISCCCE) using an online survey of 59 CHC staff. Of 38 respondents (64.4% response rate), most perceived their engagement positively, with over 92% feeling respected by ISCCCE collaborators and perceiving projects as beneficial. Limited staff time and resources were the main challenges identified. This study suggests the utility of gathering feedback to evaluate community research engagement and inform adaptations of research processes to optimize partnership quality.

## Introduction

Community engagement in research is widely regarded as essential to inform relevant research questions and study design, facilitate translation of evidence into practice, and advance health equity [1–3]. Expert groups have defined core principles of community engagement, including the 9 Principles of Community Engagement developed by the Clinical and Translational Science Awards (CTSA) Consortium’s Community Engagement Key Function Committee Task Force (Table 1) [1,4]. However, conducting community-engaged research can present challenges to both academic researchers and community members [2], including community-based clinicians and health facility staff. There is growing interest in developing measures to evaluate the degree to which research activities align with community engagement principles [5–8]. Nonetheless, community engagement remains infrequently measured in research projects. This limits opportunities to understand gaps, make data-driven adaptations, and identify strategies to successfully operationalize community engagement principles.

**Table 1.** Operationalization of community engagement principles in ISCCCE implementation research projects (adapted from Kruse et al^1^)

Community-engaged research principles (adapted from the CTSA consortium’s community engagement key function committee task force^2^)	Operationalization in ISCCCE implementation research projects and related survey questions to evaluate success
1. **Be clear** about the purposes or goals of the engagement effort and the populations and/or communities you want to engage	- MLCHC’s role as key I-Lab partner and connector to CHCs was defined prior to grant submission. *Related survey question: -“Please choose the extent to which you agree or disagree with the statements on the left: “For this pilot, I am clear about the following components as outlined in the project description (data collection, participation in meetings, attendance at Implementation Learning Community meetings, availability of the virtual learning platform)”*
2. **Become knowledgeable** about the community’s culture, economic conditions, social networks, political and power structures, norms and values, demographic trends, history, and experience with efforts by outside groups to engage it in various programs. Learn about the community’s perceptions of those initiating the engagement activities	- MPI had prior collaboration with MLCHC and understood its structures and priorities. - MPI and several co-investigators had extensive familiarity with CHC communities, populations, and cancer prevention needs - MLCHC provided on-going information about operating conditions of CHCs, priorities, and competing demands, concurrent initiatives, and opportunities to engage with CHC staff and leadership to develop first-hand knowledge and relationships. - MLCHC and the Implementation Learning Community provided opportunities for bi-directional learning and feedback -Investigators learned directly from CHC partners about their context and resources, in the process of partnering in pilot studies. -Yearly community engagement surveys actively gathered CHC teams’ impressions of ISCCCE collaboration. *Related survey questions:* *-“Please choose the extent to which you agree or disagree with the following statements: “I feel respected by the ISCCCE team for my knowledge and contributions,” “The ISCCCE team was sensitive to the needs of our patient population,” “The ISCCCE project team was sensitive to the constraints and realities of my health center.””* *-“Please choose the extent to which you agree or disagree with the statements on the left: “The following project components* ***were reasonable to carry out*** *at our health center (data collection, participation in meetings, attendance at Implementation Learning Community meetings, availability of the virtual learning platform).”*
3. Go to the community, **establish relationships, build trust,** work with formal and informal leadership, and seek commitment from community organizations and leaders to create processes for mobilizing the community	- Community partners” input on resources for CHCs suggested significant funding be provided, which was included in budget, with trade-offs to the science team. - Community partners’ input on how to support and engage CHCs was integrated into infrastructure and study design. -Offering flexibility and asking questions that seek to understand CHC strengths and resources constraints acknowledged CHC challenges and build trust. - MLCHC is a trusted partner that engages CHC leadership & staff in ISCCCE activities. *Related survey questions: “Please choose the extent to which you agree or disagree with the following statements: “I feel respected by the ISCCCE team for my knowledge and contributions.””*
4. Remember and accept that collective self-determination is the responsibility and rights of all people in a community. No external entity should assume it can bestow on a community the power to act in its own self-interest **(Community empowers itself)**	- Partner CHCs determined if they wished to participate in research projects; an open call for all opportunities ensured self-selection and access. - MLCHC provided input on all study ideas; projects were not pursued if MLCHC did not think they were viable or relevant to the CHCs. *Related survey questions: “Please choose the extent to which you agree or disagree with the following statements: “I feel that the research outcomes will benefit our health center and patients.””*
5. **Partnering with the community is necessary** to create change and improve health	- MLCHC was actively engaged in all decisions about pilot funding and research activities. - MLCHC helped drive implementation activities and ensured efficient integration into the workflow. -During project implementation, the I-Lab’s partnership with CHCs extended to modifications outside of the core activities, where scientifically appropriate and when aligned with CHC health goals. - Research projects were designed to help CHCs gather needed information from their community partners, and to develop strategies to move that knowledge into action.
6. All aspects of community engagement must **recognize and respect the diversity of the community**. Awareness of the various cultures of a community and other factors affecting diversity must be paramount in planning, designing, and implementing approaches to engaging a community	- ISCCCE embraced diversity on many levels. From a disciplinary perspective, ISCCCE centered our community partners’ input wherever possible, and brought MLCHC partners into the leadership team and MPI roles. - Leadership and staff in the CHCs often reflect the racial/ethnic diversity of the communities that they serve, which builds our understanding and awareness of community culture and needs. -CHC partners work with investigators to determine how core elements of interventions or implementation strategies can be modified or adapted to achieve fidelity while optimizing the fit in their context. *Related survey questions: “Please choose the extent to which you agree or disagree with the following statements: “The ISCCCE team was sensitive to the needs of our patient population,” “I feel respected by the ISCCCE team for my knowledge and contributions.””*
7. Community engagement can only be sustained **by identifying and mobilizing community assets and strengths and by developing the community’s capacity** and resources to make decisions and take action	- Most ISCCCE pilots use a population management platform that is shared by the 32 participating CHCs. This allows us to build strategies and tools that can be sustained. - The I-Lab created mechanisms for capacity-building related to implementation and two-way engagement with the study team. - ISCCCE actively engaged partners in grant-writing, presentations and manuscripts to build their capacity and to enrich our understanding of the findings. - ISCCCE provided mentoring and access to national training opportunities for CHC staff that wish to expand their research skills. - ISCCCE engaged partners as co-investigators on studies where there is interest. *Related survey questions:* *- “To what extent does involvement in publications increase your interest to participate in research projects?”* *-“Please choose the extent to which you agree or disagree with the following statements: “The resources provided for the project were sufficient to support our participation in the pilot.””* *-“What factors helped your CHC to implement this ISCCCE project?”* *-“What barriers did you face in implementing this ISCCCE project, whether internal (e.g. lab interface) or external factors (e.g. reimbursement)?”*
8. Organizations that wish to engage a community as well as individuals seeking to effect change must be prepared to **release control of actions or interventions to the community and be flexible enough to meet its changing needs**	- Survey evaluation of community engagement was led by MLCHC in order to ensure unbiased feedback. - Resources provided to CHCs participating in implementation studies were designed to be used as flexibly as possible. - A major part of ISCCCE’s work was designing an adaptation process that allows CHCs to make changes to their implementation activities to address their priorities, needs, and resources. - Investigators re-designed and modified studies when emerging needs dictate a shift (e.g. the pandemic), as warranted by CHC interest. *Related survey questions:* *-“Please choose the extent to which you agree or disagree with the following statements: “The ISCCCE project team was sensitive to the constraints and realities of my health center.””*
9. Community collaboration requires **long-term commitment** by the engaging organization and its partners	- ISCCCE provided expertise in establishing a research infrastructure to the MLCHC, which has developed its own internal research capacity - ISCCCE routinely met with partners to identify new sources of funding and make joint decisions as to which opportunities to pursue.

1 Kruse GR, Lee RM, Aschbrenner KA, et al. Embedding community-engaged research principles in implementation science: the implementation science center for cancer control equity. *Journal of Clinical and Translational Science.* 2023;7(1):e82.

2 CTSA Community Engagement Key Function Committee Task Force. Principles of Community Engagement. Task Force on the Principles of Community Engagement. https://www.atsdr.cdc.gov/communityengagement/pdf/PCE_Report_508_FINAL.pdf. Published 2011. Accessed January 7, 2025.

ISCCCE = Implementation Science Center for Cancer Control Equity. MLCLC = Massachusetts League of Community Health Centers. I-Lab = Implementation Laboratory.

The Implementation Science Center for Cancer Control Equity (ISCCCE) was one of seven Implementation Science Centers funded by the National Cancer Institute from 2019–2024. ISCCCE was a partnership among the Harvard T.H. Chan School of Public Health, the Kraft Center for Community Health at Massachusetts General Hospital, and the Massachusetts League of Community Health Centers (MLCHC). A core component of ISCCCE’s model entailed collaborative pilot research projects engaging academic researchers and community health center (CHC) clinicians and staff [9]. MLCHC played a critical role in ISCCCE leadership and was the primary liaison between academic partners and the CHCs. ISCCCE’s Implementation Laboratory (I-Lab) led research capacity-building activities and supported pilot project implementation at CHCs.

ISCCCE sought to embed in its work the 9 CTSA Principles of Community Engagement by articulating ways in which each principle would be represented in our work processes (Table 1). For example, we operationalized principle 7, “identify and mobilize community assets and strengths and by develop the community’s capacity and resources” in several ways in pilot project planning, including: (1) using a population management platform shared by the 32 participating CHCs as the basis of data collection; (2) actively engaging partners as co-authors on presentations and manuscripts to build their capacity and to enrich understanding of the findings; and (3) engaging interested CHC partners as co-investigators on studies [9]. To evaluate the degree to which ISCCCE adhered to these principles and inform continuous improvement in engagement processes, MLCHC annually surveyed all research pilot participants from CHCs. This manuscript describes the results of this evaluation to provide insight into the successes and limitations of ISCCCE’s approach to community-engaged research. We anticipate that ISCCCE’s evaluation process may be a useful example for the growing number of institutions seeking to adopt and evaluate community-engaged approaches.

## Materials and methods

### Implementation research projects

Eight pilot implementation research projects were sponsored by ISCCCE from 2020–2024 to support CHCs’ adoption of evidence-based practices in cancer prevention and control, build sustainable implementation and research capacity at CHCs, and address cancer inequities in Massachusetts. From 2020–2023, each pilot was led by an academic investigator and involved 2–4 CHCs that chose to participate and received pilot funding to facilitate their participation. In 2023–2024, ISCCCE funded 2 pilot projects that were initiated and led by CHC-based investigators and implemented by CHC-based teams with ISCCCE administrative and methodological support as needed.

Example topics included bundling colorectal cancer screening outreach with social risk assessment [10] and piloting implementation strategies to facilitate smoking cessation and lung cancer screening. CHC staff played a range of roles – they co-developed the research projects, led adaptation of projects to their facilities’ and communities’ contexts [11], and oversaw implementation; they facilitated data collection, attended team meetings, presented results internally to other CHCs and externally e.g. at conferences, co-authored manuscripts, and partnered in additional funding applications. CHC staff were also encouraged to attend quarterly Implementation Learning Community (ILC) meetings. Organized by the I-Lab, the ILCs brought together CHC staff from across the state for 2-hour video conferences focused on cancer control equity, including sharing pilot project experiences. The I-Lab also developed a virtual learning platform (using Canvas) intended to facilitate communication about projects and support CHC capacity-building.

### Survey development

The 9-item survey was co-developed by ISCCCE investigators and MLCHC based on CTSA principles (Table 1). Items examined CHC staff’s understanding of and satisfaction with different pilot project components, activities, and support offered. Degree of agreement was assessed using Likert scales. Respondents were asked two open-ended questions: 1) “What factors helped your CHC implement this ISCCCE project?”; and 2) “What barriers did you face in implementing this ISCCCE project, whether internal (e.g. lab interface) or external factors (e.g. reimbursement)?” Respondents were encouraged to share additional comments and suggestions for future research topics. Surveys took 10–15 minu to complete.

### Survey recipients and administration

ISCCCE staff identified all CHC staff participating in ISCCCE-supported pilot projects.

MLCHC staff then administered the online survey via email to the identified CHC staff after completion of the pilot project period (typically one year) from 2021–2025. One to two email reminders were sent to non-responders.

### Analysis

MLCHC shared pilot-level aggregate data from surveys with ISCCCE investigators. The ISCCCE team used descriptive statistics to analyze the quantitative data. For Likert scales, “agree” and “strongly agree” categories and “disagree” and “strongly disagree” were combined. Qualitative data from free-text responses were reviewed by 2 investigators and coded using a rapid qualitative approach with an Excel spreadsheet organized by survey question [12]. Coders summarized key takeaways for each question and collaboratively identified primary themes.

### Ethics

The project was determined to be Not Human Subjects Research by the Harvard Longwood Campus Office of Regulatory Affairs and Research Compliance.

## Results

### Respondents

Surveys were sent to 59 pilot participants; 38 (64.4%) responded. Respondents represented a variety of CHC roles, including CHC leadership, quality improvement staff, clinicians, medical assistants, patient navigators, and lab personnel (Appendix Table).

### Quantitative results

Table 2 shows quantitative survey results. Respondents were enthusiastic about the potential benefits of the research projects and the ISCCCE team’s respectfulness, contextual awareness, and flexibility (Table 2). Ninety-five percent of respondents agreed the research outcomes would benefit their CHC and patients, and 92.1% felt respected for their knowledge and contributions. Similarly, 86.8% felt the ISCCCE team was sensitive to their CHCs’ constraints and realities, 89.5% felt ISCCCE was sensitive to patient needs, and 86.8% would be enthusiastic about future ISCCCE research participation. Most (81.6%) felt resources were sufficient to support pilot participation. When asked whether they were clear about data collection requirements, 81.6% of participants agreed. Similarly, most respondents reported clear expectations for meeting participation (84.2%) and attendance at the ILC (81.6%). In contrast, only 60.5% were clear on availability of the Canvas virtual learning platform. When asked whether project components were reasonable to carry out at their CHC, 84.2% agreed; 89.5% felt that research meeting participation was reasonable, and 86.8% felt ILC participation was reasonable. In contrast, 50.0% felt that Canvas was reasonable to use. With regard to the utility of specific activities, team meetings were felt to be useful to a great extent for 68% of participants, and ILC participation for 42.1%. However, only 18.4% of respondents felt the Canvas virtual platform was useful. Most respondents were enthusiastic about participating in publications, with 45.2% noting that publication involvement increased their interest in project participation “to a great extent,” and 32.3% noting it increased their interest “somewhat.”

**Table 2.** Community health center staff responses to community engagement survey, 2021–2025 (*N* = 38)

Survey question	Agree or strongly agree, *n*(%)	Neutral, *n*(%)	Disagree/Strongly disagree, *n*(%)	Missing, *n*(%)
**For this pilot, I am clear about the following components as outlined in the project description**				
Data collection requirements	31 (81.6)	5 (13.2)	1 (2.6)	1 (2.6)
Participation in meetings with the ISCCCE research team	32 (84.2)	4 (10.5)	1 (2.6)	1 (2.6)
Attendance at implementation learning community meetings	31 (81.6)	4 (10.5)	2 (5.3)	1 (2.6)
Availability of the virtual learning platform, canvas	23 (60.5)	8 (21.0)	6 (15.8)	1 (2.6)
**The following project components were reasonable to carry out at our health center.**				
Data collection requirements	32(84.2)	2 (5.3)	3 (7.9)	1 (2.6)
Participation in meetings with the ISCCCE research team	34 (89.5)	2 (5.3)	1 (2.6)	1 (2.6)
Attendance at implementation learning community meetings	33 (86.8)	3 (7.9)	1 (2.6)	1 (2.6)
Availability of the virtual learning platform, canvas	19 (50.0)	13 (34.2)	3 (7.9)	3 (7.9)
**Please tell us the extent to which you agree or disagree with the following statements.**				
I feel that the research outcomes will benefit our health center and patients	36 (94.7)	0 (0.0)	1 (2.6)	1 (2.6)
I feel respected by the ISCCCE team for my knowledge and contributions	35 (92.1)	1 (2.6)	1 (2.6)	1 (2.6)
The ISCCCE project team was sensitive to the constraints and realities of my health center	33 (86.8)	3 (7.9)	1 (2.6)	1 (2.6)
The ISCCCE team was sensitive to the needs of our patient population	34 (89.5)	2 (5.3)	1 (2.6)	1 (2.6)
I would be enthusiastic about participating in another research study with ISCCCE	33 (86.8)	2 (5.3)	2 (5.3)	1 (2.6)
The resources provided for the project were sufficient to support our participation in the pilot	31 (81.6)	4 (10.5)	2 (5.3)	1 (2.6)

	To a great extent, *n*(%)	Somewhat, *n*(%)	Very little/Not at all, *n*(%)	I have not participated in this support, *n*(%)	Missing
**How useful have the following activities been to you in participating in the project?**					
Meetings with the ISCCCE project team	26 (68.4)	10 (26.3)	0 (0.0)	1 (2.63)	1 (2.6)
Learning community meetings that include other health centers	16 (42.1)	8 (21.0)	1 (2.6)	12 (31.6)	1 (2.6)
Virtual learning platform, canvas	7 (18.4)	8 (21.0)	3 (7.9)	19 (50.0)	1 (2.6)
To what extent does involvement in publications increase your interest to participate in research projects? (*n* = 31*)					
	14 (45.2)	10 (32.3)	6 (19.3)	N/A	1 (3.2)

* This question was only included in surveys in years 2–5.

### Qualitative results

Themes identified in free text responses are shown in Table 3. Among successes of the collaboration noted by CHC participants, participants felt that their relationship with ISCCCE was positive and built on shared goals, with the ISCCCE team demonstrating flexibility and supportiveness. A second notable theme was that collaboration fostered an interest among CHC staff in being engaged in research in the future. Consistent with the quantitative results, respondents were enthusiastic about participating in publications and increasing CHC visibility. Participants described internal challenges to implementing the research projects at their CHCs, including competing priorities, limited clinician time, and limited resources (e.g. fecal immunochemical kit shortages for colorectal cancer screening). External factors such as the COVID-19 pandemic and cancer screening guideline changes presented additional challenges. Finally, respondents noted the difficulty of completing a project in a one-year timeframe, including due to administrative delays in setting up contracts with ISCCCE.

**Table 3.** Community health center staff responses to community engagement survey open-ended questions, organized by theme

Theme	Exemplar quotes
**Successes**	
Community health centers staff perceived ISCCCE staff and investigators as supportive, encouraging, flexible and helpful in advancing projects’ success.	*- “Really enjoyed this experience, felt very supported and amongst like-minded colleagues.”* *- “I found the community to be supportive and encouraging.”* *- “Flexibility & encouragement of ISCCCE pilot study team with ensuring meetings, data submissions, meetings with other stakeholders.”* *- “The biweekly meetings with the Harvard team have really helped address our concerns and troubleshoot the program.”* *- “We truly had such a positive engagement on this project.”*
Community health center staff expressed interest in working on publications and future projects.	*- “It is very empowering to be invited to participate in the publications. I was asked to review an abstract and my responses were thoughtfully incorporated.”* *- “This has been a wonderful experience. I would love to get involved further. It helps us to increase the exposure of our health center.”* *- “We would definitely work with ISCCCE in the future for other pilot studies.”* *- “We would like to participate in more research projects but are limited by the size of our staff and providers to allow the time to what we would like to do.”*
**Challenges**	
Limited community health center staff time, competing priorities and structural challenges were the primary barriers to project implementation.	*- “Just too many competing priorities”* *- “FIT kit shortage”* *- “Internal staffing shortages”* *- “Carving out provider time.”* *- “The new EHR system has really impacted our ability to operate the program.”* *- “We still have more implementation components to complete. The paucity of time, funding, and lean teams at our health center contributed to barriers. Time and staffing shortages.”*
External factors such as Covid-19 and changes in guidelines impacted pilot implementation.	*- “Competing priorities with COVID Testing/Vaccines, lack of available staff to help implement workflows or conduct outreach.”* *“change in colorectal cancer screening measure to include age 45-50”*
One-year pilot timeline was challenging for community health centers.	*- “Internal administrative hurdles created impactful delays given the 12-month project.”* *- “We had internal barriers that had nothing to do with the research project that delayed implementation of the contract and payment invoicing.”* *- “Short timeline for implementation, ideally would have had more time to gather more feedback.”* *- “Our planned activities were updated to accommodate a shortened timeline.”* *- “Timeline was short so we had to cut down on number of focus groups as well as utilize a rapid qualitative analysis.”* *- “Too short of time span”*

## Discussion

In this evaluation of pilot research projects entailing collaboration between academic investigators and CHC-based investigators and staff, CHC participants perceived their engagement positively. Most respondents felt that the collaboration adhered to community engagement principles in terms of ensuring clarity about and feasibility of project requirements, fostering trusting and respectful relationships between academic and CHC collaborators, supporting projects with benefits for communities and CHCs, and developing CHCs’ capacity for and interest in research.

Staff responses have helped ISCCCE prioritize activities that fostered positive engagement. The activity that the most respondents felt was useful were meetings with the ISCCCE team, suggesting that despite CHC staff’s inherent time constraints, meetings fostered collaboration and were felt to be productive. There was also high enthusiasm for the ILC, which brought together CHC staff from around the state. As a result, the ILC has been sustained after the end of ISCCCE’s grant funding through our CTSA Community Engagement Program. Publication interest was also notably high, encouraging investigators to prioritize inclusion of CHC partners in manuscript development. Over 90% of center publications had CHC partner co-authors [13]. CHC staff were less enthusiastic about the Canvas virtual platform initially envisioned as a communication tool, likely due to competing demands on their time and use of multiple other online systems for routine work. Thus, after 2 years, ISCCCE staff adapted the platform to be primarily a repository for study-related materials rather than a communication/ collaboration tool.

The most frequently identified barriers to project implementation were CHCs’ internal barriers, including staff time, and external factors such as the COVID-19 pandemic. Flexibility was built into ISCCCE’s structure and research processes, allowing investigators and CHC partners to collaboratively adapt projects in response to these inner and outer context factors [11]. Respondents also noted administrative challenges to operationalizing new contracts, despite ISCCCE’s deliberate efforts to streamline contracting processes. Projects with longer time horizons (>1 year) may be more feasible in settings that have burdensome research or financial approval processes and/or limited administrative staff.

This evaluation has several strengths. First, it contributes to a small literature evaluating the success of community engagement in research. Second, the evaluation survey was co-developed by community and academic partners and administered by community partners, who owned the data. This maximized questions’ relevance and helped the team adapt approaches in response to findings. Having MLCHC administer the survey may also have minimized acceptability bias and promoted participation. However, our evaluation has limitations. First, we did not formally validate the survey tool. A validated tool to assess stakeholder engagement, the Research Engagement Survey Tool (REST), was published in 2022, 3 years into our evaluation process, and has similarities with our approach. For example, REST is founded on community engagement principles and includes questions about respect for community partners and projects’ community benefits. The REST tool focuses on general adherence to the principles and was designed for a broad array of partners rather than specifically to CHCs [4], while our tool elicited concrete feedback about specific activities and suggestions for improvement. Combining these approaches could be valuable in the future, as long as tools remain concise and easy for community partners to complete. Second, our sample size was small, limiting the extent to which we could examine associations of specific engagement strategies with perceived engagement. Third, non-response bias may have affected our results, though our response rate of 64.4% was higher than many recent surveys of health care professionals [14]. Finally, we surveyed participants at the end of one-year projects. More frequent assessments could allow more timely adaptations during project implementation. Reassessments over a longer time horizon would help evaluate the durability of a partnership.

In conclusion, this evaluation of an academic-CHC research partnership demonstrates the value of deliberate investment in operationalizing community engagement principles to facilitate partnerships’ success. It also underscores the importance of evaluating the degree to which research adheres to these principles in order to continually improve these relationships. In turn, productive partnerships may enhance the feasibility, acceptability, and effectiveness of research interventions.

## Supporting information

Pace et al. supplementary material Pace et al. supplementary material

## Supplementary material

The supplementary material for this article can be found at https://doi.org/10.1017/cts.2025.86.
