# Supplementary material for: Evaluating the success of community engagement in the Implementation Science Center for Cancer Control Equity
Source: J Clin Transl Sci. 2025 May 16;9(1):e122. doi: 10.1017/cts.2025.86 (PMC12209956; doi:10.1017/cts.2025.86)
Supplement: Pace et al. supplementary material [file S205986612500086Xsup001.docx]

**Appendix Table 1. Community health center survey respondent roles (N=38)**

| Role | n (%) |
| --- | --- |
| Leadership | 4 (10.5) |
| Population health/ quality staff | 17 (44.7) |
| Clinician | 8 (21.0) |
| Medical assistant | 2 (5.3) |
| Patient navigator/outreach worker | 3 (7.9) |
| No response | 1 (2.6) |
| Other | 3 (7.9) |
